# Theta Modulated Neural Phase Coherence Facilitates Speech Fluency in Adults Who Stutter

**DOI:** 10.3389/fnhum.2019.00394

**Published:** 2019-11-19

**Authors:** Ranit Sengupta, J. Scott Yaruss, Torrey M. Loucks, Vincent L. Gracco, Kristin Pelczarski, Sazzad M. Nasir

**Affiliations:** ^1^Department of Communication Sciences and Disorders, Northwestern University, Evanston, IL, United States; ^2^Department of Communicative Sciences and Disorders, Michigan State University, East Lansing, MI, United States; ^3^Department of Communication Sciences and Disorders, Faculty of Rehabilitation Medicine, University of Alberta, Edmonton, AB, Canada; ^4^Institute for Stuttering Treatment and Research, Faculty of Rehabilitation Medicine, University of Alberta, Edmonton, AB, Canada; ^5^Haskins Laboratories, New Haven, CT, United States; ^6^School of Family Studies and Human Services, Kansas State University, Manhattan, KS, United States; ^7^Indiana Academy, Ball State University, Muncie, IN, United States

**Keywords:** neural communication, speech, phase coherence, motor control, stuttering

## Abstract

Adults who stutter (AWS) display altered patterns of neural phase coherence within the speech motor system preceding disfluencies. These altered patterns may distinguish fluent speech episodes from disfluent ones. Phase coherence is relevant to the study of stuttering because it reflects neural communication within brain networks. In this follow-up study, the oscillatory cortical dynamics preceding fluent speech in AWS and adults who do not stutter (AWNS) were examined during a single-word delayed reading task using electroencephalographic (EEG) techniques. Compared to AWNS, fluent speech preparation in AWS was characterized by a decrease in theta-gamma phase coherence and a corresponding increase in theta-beta coherence level. Higher spectral powers in the beta and gamma bands were also observed preceding fluent utterances by AWS. Overall, there was altered neural communication during speech planning in AWS that provides novel evidence for atypical allocation of feedforward control by AWS even before fluent utterances.

## Introduction

Stuttering is a speech production disorder involving the central nervous system, but the specific neurological basis is still unclear. Research suggests a genetic component (Shugart et al., 2004; Riaz et al., 2005; Jones et al., 2012; Nouri et al., 2012) may alter early brain structural development related to the speech production system (Sommer et al., 2002; Beal et al., 2007, 2013, 2015; Chang et al., 2008; Connally et al., 2014; Misaghi et al., 2018). Although this may impact sensorimotor and linguistic processing, there is a lack of understanding of how these structural differences affect the neural processes that underlie fluent vs. stuttered speech. Prior work has proceeded along two different lines (Belyk et al., 2015, 2017; Connally et al., 2018): one to identify state differences (reflecting differences associated with the moment of stuttering itself), and the other to identify trait differences [reflecting differences in people who stutter that distinguish between adults who stutter (AWS) and adults who do not stutter (AWNS) regardless of speech fluency]. In order to obtain information on the neural processes associated with either fluent or stuttered speech, an approach that can capture the dynamics of speech production is needed. In a previous study from this lab, neural phase coherence was found to be useful in assessing pre-speech neural activity in individuals who do not stutter (Sengupta et al., 2016). Communication within functional brain networks in humans is thought to be accomplished by neural phase coherence, reflecting synchronous firing of neuronal populations in goal-directed tasks such as speech production (Fries, 2005, 2015; Schroeder et al., 2008; Arnal et al., 2011; Varela et al., 2011; Mercier et al., 2015). It is hypothesized that there is a dis-integration in speech motor planning, as evidenced by a reduction in neural phase coherence within the speech production system that precedes stuttering disfluencies (Loucks and De Nil, 2006; Sengupta et al., 2017).

Evidence for this hypothesis comes, in part, from neuroimaging studies that have identified differences in brain regions of AWS under conditions that focus on the preparatory phase of speech production (Salmelin et al., 2000; Chang et al., 2009). A growing number of electroencephalography (EEG) and magnetoencephalography (MEG) studies tracking neural activity associated with speech production in AWS have revealed aberrant dynamics during speech planning activity in the beta band of AWS (Sengupta et al., 2017), as well as differences in evoked potentials and neural oscillations related to the fluent speech of AWS (Beal et al., 2010, 2011; Daliri and Max, 2015; Vanhoutte et al., 2015; Mersov et al., 2016). Suppression of beta power during the planning phase of overt speech is well-known (Hebb et al., 2012), and there is also emerging evidence that beta band motor activity is suppressed in the speech of those who stutter (Salmelin et al., 2000; Mersov et al., 2016). However, these studies have not assessed how coherence across the neural bandwidths contributes either to stuttering or fluency.

In a prior study from this lab, it was shown that AWS exhibit reduced sensorimotor adaptation during vowel production that was accompanied by aberrant neural phase coherence in theta-gamma bands compared to AWNS (Sengupta et al., 2016). In particular, another study from this lab showed that prior to the onset of stuttering, neural phase coherence in the gamma band increased in the frontal part of the scalp (Sengupta et al., 2017). This finding, which is consistent with neural overactivation, constitutes evidence for dysfunction in brain wave oscillations and offers potential for identifying the actual brain state that precedes moments of stuttering. The present study compliments the previous finding by testing whether neural coherence varies preceding fluent production of single words by AWS. Altered coherence during the planning of fluent utterances would suggest that the trait of stuttering is characterized by a core difference in how the speech motor network is coordinated. It should be noted that, although the sample in this study the same as the sample as in the previous study (Sengupta et al., 2017), the focus of the study is on the comparison of the fluent speech between AWS and AWNS. Moreover, the previous study did not include any analysis of the fluent speech in AWNS.

Overall, this study critically examines whether fluent speech behavior in AWS involves anomalous patterns of neuronal phase coherence prior to speech onset and provides a proof of principle for this approach, despite a relatively small sample size. Such anomalies, as measured by phase coherence between EEG frequency bands, reflect miscommunication within the speech motor network. Specifically, the beta band is expected to contribute to the trait of stuttering, due to its involvement in speech planning. Moreover, since theta and gamma bands are implicated in motor adaptation and motor memory, contributions from these bands are expected also to play a major role in understanding how fluent speech is produced by AWS.

## Materials and Methods

### Participants

Participants included eight AWS [2F (females); 26 ± 1.3 years; mean and SE] with persistent stuttering and eight AWNS (3F; 22 ± 1.2 years). All participants were native English speakers with no known history of hearing or neurological disorders (other than stuttering). Participants received compensation for their participation. Stuttering frequency, assessed according to Systematic Disfluency Analysis (Gregory et al., 2003), ranged from 8.5% to 24%, with a mean of 15.4%. It should be noted that the chosen AWS participants are inhomogeneous in stuttering severity and for a better interpretation of the reported results more homogeneous samples need to be tested (see “Discussion” section below). All experimental procedures were approved by Northwestern University’s Institutional Review Board (IRB), and written informed consent was obtained from all participants. The experiments were performed in accordance with the relevant guidelines and regulations set by Northwestern IRB (adhering to Helsinki Declaration). An earlier article (Sengupta et al., 2017) focused only on the AWS for investigating the cortical state of disfluency.

### Stimuli and Experimental Setup

The stimuli set included a list of 80 complex and multisyllabic target speech tokens (2–6 syllables long). Five of the tokens were real words, while others were nonwords that were either distorted slightly to form “word-like” nonwords (e.g., teslivision; 34 in total) or “less word-like” nonsense words (e.g., malubaishoi; 41 in total). The stimuli contained a majority of non-words in order to reduce word-familiarity. Phonotactic probability for both the real and nonwords was roughly equivalent (low frequency, like nonwords) and both stimuli sets produced qualitatively similar level of fluency.

**Figure 1:**
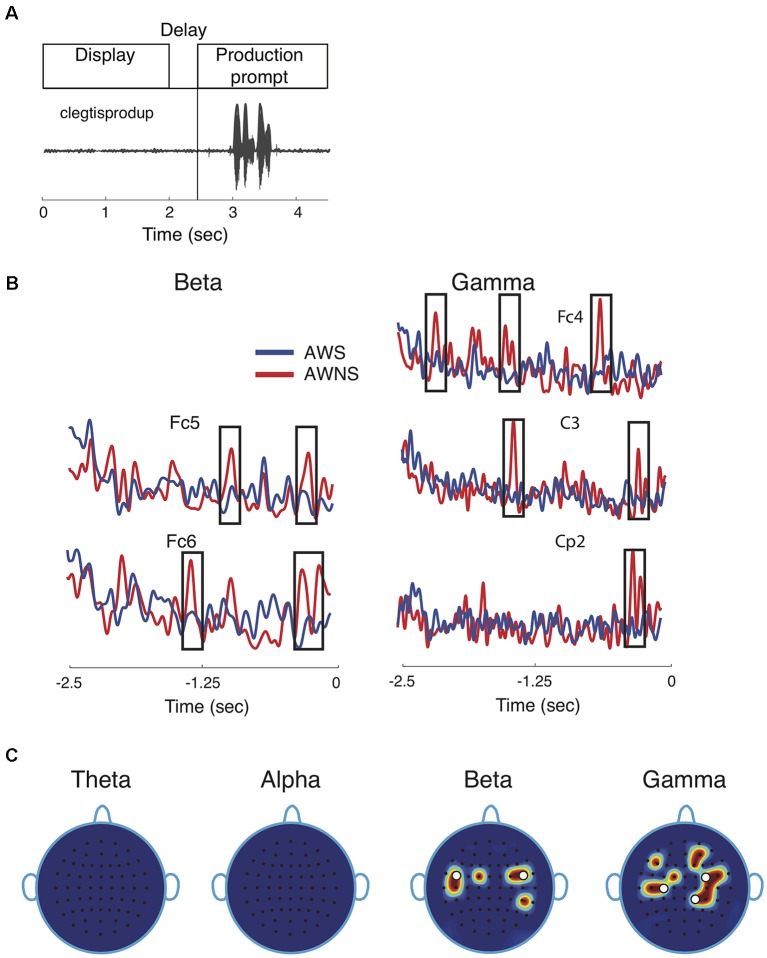
Speech task and spectral power results. **(A)** Participants performed a delayed reading task in which target utterances were displayed for 2 s, followed by a prompt to read the displayed utterance aloud after a 0.5 s delay. The speech waveform corresponding to the utterance “clegtisprodup,” is shown. **(B)** Representative power traces for beta and gamma band are shown for adults who stutter (AWS; blue) and adults who do not stutter (AWNS; red). The 2.5-s portion of the power trace from the start of the word display to the appearance of the production prompt is shown at 0 s. Significant differences are shown to occur in time windows marked by rectangular windows. **(C)** Scalp electrode locations display significant power differences between the two groups. Effects were observed for beta band at electrode locations Fc1, Fc5, C5, Fc2, Fc4 and Cp4 and for gamma band at Fc1, C3, C5, Fc4, Fc6, C4, Cp2, Cp4, F5, F2, and Af4. White circles denote electrode locations that showed differences in phase coherence.

The speech task involved reading aloud the target tokens while EEG signals were being continuously recorded from the scalp (Figure 1A). The tokens were displayed for 2 s. After a 0.5 s delay, a plus sign appeared (production prompt) that cued participants to read the word immediately and aloud (within 2 s). Cues regarding meaning or correct pronunciation were not provided. A real-time Labview system (National Instruments) was used to display the speech tokens. The 80 speech tokens were each repeated five times in groups of 40 blocks.

All experiments took place in a soundproof booth, and microphone output (Sennheiser ME-66) was recorded at 40 KHz. Each utterance was checked for the presence of disfluencies (specifically, part-word repetitions, prolongations, or blocks) by a speech-language pathologist with expertise in fluency disorders. Trials in which the stimuli were uttered before the prompt signal and those whose initiation exceeded the 2 s prompt window were discarded from the analyses (2.6% of all the trials). For each participant, a fluency score (whether a single speech token is fluent or not) was obtained by taking the percentage of fluent utterances over all trials. The mean fluency score on the single words produced in the experiment was 90.0 ± 2.9% (mean and SE) for the AWS and 98.7 ± 0.1% for the AWNS. This high rate of fluency is common for AWS on single-word productions.

### EEG Acquisition and Pre-processing

A 64-channel Brainvision system was used to record EEG data at 512 Hz. The electrodes mounted on the scalp followed the standard 10–20 system, and the electrical impedances were kept below 10 kΩ. For the analyses reported here, electrodes over the occipital region, as well as electrodes over the extreme temporal and frontal regions, were excluded to reduce motion artifacts. Subsequent analysis involved 38 electrodes over the temporal, parietal, and frontal areas of the scalp (Sengupta et al., 2016, 2017). Participants were instructed to minimize eye blinks and head movements during word production. Brief pauses between trials and between blocks were provided to minimize fatigue and muscle tension. The real-time Labview system delivered a transistor-transistor logic (TTL) pulse at the moment of the stimulus display and also at the production prompt. These were used in subsequent offline analyses to align EEG signals with the spoken utterances.

The EEG signals were band-pass filtered offline between 0.75 and 55 Hz using a 2nd order Butterworth filter (EEGLAB toolbox, Delorme and Makeig, 2004). All trial event-related potential (ERP) epochs were aligned at the onset of production prompt and re-referenced at electrode Afz (Jarmolowska et al., 2013; Sengupta and Nasir, 2015). It should be noted that average reference was not used since the analyses did not include all scalp electrodes. The analyses reported in this article include a time window of 2,500 ms preceding the production prompt.

Stereotypical artifacts caused by muscular activity were removed by discarding epochs in which the scalp voltage at any of the electrode locations exceeded 75 μV. As a basis for further artifact rejection, the presence of aberrant temporal patterns and large negative kurtosis were detected (Sengupta and Nasir, 2015). Muscle artifacts were eliminated by detecting spectral peaks that coincided with muscle activation. Automated techniques based on independent component analysis (as implemented in EEGLAB toolbox, Delorme and Makeig, 2004) were used for artifact detection. About 15% of the trials were discarded due to artifact rejection. It should be noted that artifacts could also arise due to electromagnetic interference. In this study, however, no faraday cage was used to limit this type of artifacts.

### Analysis of Neural Oscillations

Each trial epoch was first normalized by dividing it by the overall power. The normalized trials were then filtered using a 4th-order Butterworth filter to obtain the instantaneous power over four EEG frequency bands, which were theta (3–8 Hz), alpha (8–14 Hz), beta (14–30 Hz) and gamma (30–50 Hz). Instantaneous signal amplitude within each band was obtained using the Hilbert transformation, the square of which provides the instantaneous power. Neural phase coherence (Perfetti et al., 2011; Sengupta and Nasir, 2015) between lower frequency bands (theta and alpha) and higher frequency bands (beta and gamma) was computed by quantifying the degree of phase-locking between the two bands (custom-written Matlab scripts). Following the method proposed by Cohen (2008), a critical component in the computation of phase coherence is determining whether higher frequency power spectrum has a peak that is within the lower frequency range. Neural phase coherence was finally expressed as a number between 0 (perfect dyssynchrony) and 1 (perfect synchrony). Thus, phase coherence was evaluated for theta-beta and theta-gamma band pairs, and likewise for alpha-beta and alpha-gamma bands.

The phase-locking was computed using a 3 Hz long sliding frequency-window and a 400 ms long time-window that contained about 2–3 theta cycles and about 5 alpha cycles. The time-frequency spectrogram associated with neural phase coherence shows time on the horizontal axis and the upper-band frequencies (beta and gamma) on the vertical axis. The power time series of the higher frequency was first used to compute its instantaneous phase using the angle of its Hilbert transformation. Similarly, the instantaneous phase of the lower band signal was obtained using Hilbert transformation. Phase coherence between these two frequency bands was then computed for a given time window by taking the difference between their respective phase time series.

It should be noted that the computations of phase coherence can be impacted by spectral correlations present in the signal (Aru et al., 2015). Although using a fixed frequency-window may bias phase coherence analyses, the choice of small frequency-windows for the low-frequency bands, as done here, could mitigate the issue. Also, in order to ensure that filtering edge effects did not affect the computations, samples (equivalent to 20 ms) at the beginning and the end of the signal were excluded from further analysis.

### Bootstrapping and Statistical Significance

Statistical significance was obtained using bootstrap sampling techniques (Efron, 1982) after correcting for family-wise error (Pantazis et al., 2005). For each electrode, a difference *t*-score was obtained between AWS and AWNS in the following way, using custom-written Matlab scripts: for each word, the mean power (or phase-coherence) time series was calculated then averaged over all words to give the mean power (or phase-coherence) for each participant. These scores across participants (16 in total) were used to calculate the difference *t*-score (mean difference between AWS and AWNS divided by pooled standard deviation) time series at each electrode location. Next, 4,000 bootstrap samples of size 8 + 8 (shuffling AWS and AWNS) were generated using sampling methods with replacement. On each bootstrap iteration, these two samples of size 8 were used to obtain a *t*-score. Thus, there were 4,000 *t*-score time series (or time-frequency series) for each electrode. The maximum of the absolute *t-score* overall electrodes and over the entire series was then used to obtain a distribution of maximum statistics (4,000 from all bootstrap samples). The 99.5th percentile of this distribution (corresponding to *α* = 0.005) was taken as the critical *t*-score. Electrode locations for which the difference *t-score* exceeded this critical value were considered to have shown a statistically significant difference.

## Results

This study compared neural phase coherence in AWS and AWNS to resolve the pattern(s) of neural communication preceding fluent utterances. Participants were cued to read aloud target speech tokens under continuous recording of EEG brain signals from the scalp (Figure 1A). The first objective in a neural coherence study is to identify EEG frequency bands and scalp electrode locations that showed significant differences in the contrast of interest, which in this case is the group comparison of AWS and AWNS preceding fluent utterances. The power traces in beta and gamma bands from representative electrode locations are shown in Figure 1B. These were also electrode locations for which significant coherence differences were observed. The spectral power activity in AWS (blue; Figure 1B) was characterized by less pronounced peaks (marked by rectangular windows) at multiple electrode locations. This finding suggests suppression of brain activity preceding and during fluent speech (AWNS in red). No patterns in the temporal electrode locations where significant differences were observed could easily be discerned. Figure 1C displays the power scalp plots showing electrode locations with significant differences (*p* < 0.005, after correcting for familywise error; see “Materials and Methods” section). Only the higher frequency beta and gamma bands showed significant differences in power, while the lower frequency theta and alpha bands did not. Beta band activity was more localized and showed largely bilateral fronto-temporal activation at electrode locations on the left hemisphere at Fc1, Fc5, C5 and on the right hemisphere at Fc2, Fc4 and Cp4. Gamma band activity, on the other hand, was more widespread and spanned the centro-parietal regions (Fc1, C3, C5, Fc4, Fc6, C4, Cp2, Cp4) and a small part of the frontal region (F5, F2, Af4). A slight right lateral bias for both scalp regions in both groups was observed for gamma-band activity. The white dots in Figure 1C mark the electrode locations showing significant differences in neural phase coherence.

**Figure 2:**
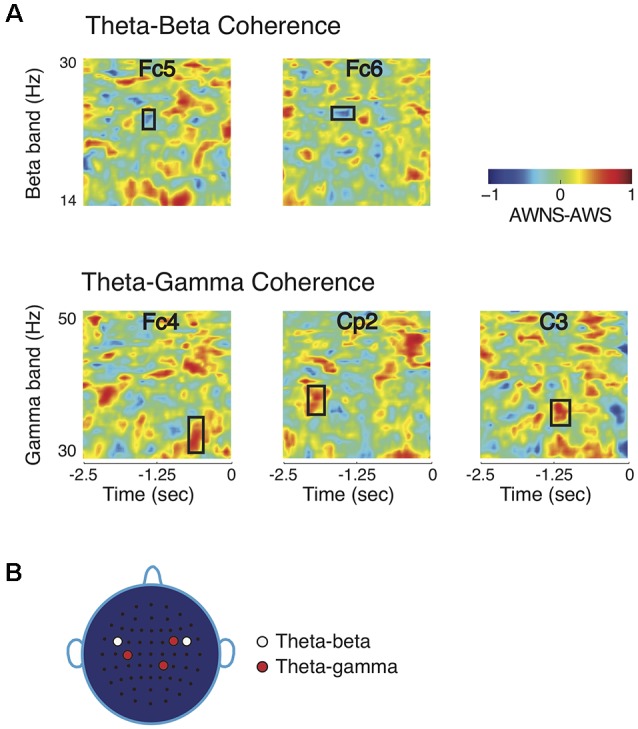
Comparisons of coherence patterns between AWS and AWNS. **(A)** Phase coherence spectrograms are scaled from −1 to 1. Only phase coherence involving theta band showed significant differences between the two groups. The ordinate of the spectrogram represents frequency range for the high-frequency beta and gamma bands, and the abscissa represent time, with 0 marking the appearance of the production prompt. Theta-beta coherence differences were observed at electrode locations Fc5 and Fc6 in time-frequency regions marked by rectangular windows, while theta-gamma differences were found at Fc4, C3 and Cp2. AWS had higher theta-beta coherence level, whereas AWNS had higher theta-gamma coherence. **(B)** Scalp electrode locations for which significant coherence differences between the two groups were observed. For theta-beta coherence, effects were observed at electrode locations Fc5 and Fc6 and for theta-gamma at Fc4, Cp2 and C3.

Noting that the higher frequency bands showed significant power differences while the lower frequency bands did not, it was then investigated whether the lower frequency bands could have a modulatory role for the beta and gamma band powers as measured by cross-frequency phase coherence. The theta band had a significant role in modulating beta and gamma bands, but alpha band did not contribute to group differences in phase coherence (Figure 2A; *p* < 0.005, after correcting for familywise error). Theta-beta phase coherence was higher in AWS than in AWNS in time-frequency regions marked by rectangular windows. The symmetrically located bilateral electrodes Fc5 on the left hemisphere and Fc6 on the right hemisphere showed significant differences in time-frequency regions centered at 24 Hz and about 1 s prior to the production prompt. On the other hand, a significantly higher theta-gamma phase coherence was observed in AWNS than in AWS at right frontal electrode location Fc4, right parietal electrode Cp2, and left central electrode C3. At these electrode locations, significant differences were observed in time-frequency windows centered, respectively, at 33, 38 and 35 Hz, and approximately 0.6, 1.9 and 1.2 s prior to the production prompt. The differences in phase coherence were thus observed at specific time-frequency windows, rather than spanning across the entire frequency range of the bands involved. The effect sizes for the observed differences were greater than 0.8 (0.81 for theta-beta coherence and 0.86 for theta-gamma coherence) providing further support to the bootstrap based analyses reported here. Lastly, the coherence scalp plot of Figure 2B summarizes the electrode locations showing significant coherence differences that preceded fluent utterances. Overall, AWS and AWNS exhibited differential phase coherence profiles between the theta-gamma and the theta-beta band pairs involving a relative increase in theta-beta coherence for AWS, while AWNS had a relative increase in theta-gamma coherence.

## Discussion

The present study examined the neural correlates of fluent speech preparation in a delayed word reading task using phase coherence. Pre-speech power differences between AWS and AWNS were observed in the high-frequency gamma and beta bands but not for alpha and theta bands. Subsequent evaluation of cross-frequency phase coherence between beta and gamma bands and the low-frequency theta band indicated significantly different coherence levels during speech planning in AWS.

These findings address the stochastic nature of disfluencies, which is a vexing problem for understanding and treating stuttering. If aberrations in the brain network that precede speech onset result in disfluencies, why are all words not affected equally? To answer this critical question, it is necessary to identify pre-speech patterns that differentiate AWS and AWNS for fluent (and disfluent) speech tokens. The absence of differences preceding fluent speech would imply that the neural activity in AWS for fluent speech is identical to AWNS and that a breakdown of such a pattern could lead to stuttering disfluencies. On the other hand, a significant group difference in neural coherence preceding fluent speech implicates a basic speech preparation anomaly (i.e., a trait difference) in AWS.

In a previous study from this lab, reduced speech motor adaptation under altered auditory feedback was observed in AWS preceding the word “head,” even in the absence of disfluency (Sengupta et al., 2016). This result pointed to a core difference in sensorimotor processing in AWS. This finding is supported by the power differences in beta and gamma bands in the AWS reported here, because these bands subserve sensorimotor function. Sustained gamma-band activity is present after the onset of visuomotor decision responses (Crone et al., 1998) and in self-paced finger movements (Ohara et al., 2000). Regarding beta activity, a large body of research suggests beta band is involved in motor planning and motor imagery (Leocani et al., 1997; Pfurtscheller et al., 1998) that could be vital for establishing communication between sensorimotor and other areas of the brain (Kilavik et al., 2013). The absence of alpha band changes prior to fluent speech onset is also an important finding in the present study. In previous studies (Sengupta et al., 2016, 2017) alpha band was found to be involved in stuttered utterances of AWS.

The phase coherence analysis revealed diverging patterns of neural communication preceding fluent utterances. Theta-beta coherence was higher in AWS compared to AWNS before speech onset, while the theta-gamma coherence was greater in AWNS. In AWS, the reduction in theta-gamma coherence level could be predicted based on the previously reported finding that theta-gamma coherence decreased during the formation of new feedforward models following speech motor adaptation for vowel production in AWNS (Sengupta et al., 2016). The decrease in theta-gamma coherence in AWS may signal that feedforward control, important for getting the speech production system in the optimal state for speech, is different from fluent controls. Stable feedforward control may play a role in facilitating fluency, while instability or disruption of feedforward control in AWS may predispose them to episodes of stuttering.

The altered preparatory phase preceding the fluent speech of AWS additionally showed increased theta-beta coherence. It has been suggested that sensory-motor integration during speech in AWS is reduced and variable, yielding an increased reliance on sensory feedback (Max et al., 2004; Loucks et al., 2012; Cai et al., 2014; Sares et al., 2018). Increased theta-beta coherence has been suggested to reflect a heightened state of sensory information processing (Engel and Fries, 2010). It is reasonable to suggest that producing complex nonwords may have elevated the need for sensorimotor integration in the AWS. In contrast, theta-gamma coherence has been suggested to form a code for representing multiple items sequentially (Lisman and Jensen, 2013). The current result from AWS during fluent speech production may be associated with a reduction in the information flow. Together, these tentative findings are consistent with a greater demand for sensory processing at the expense of up-stream networks associated with feedforward control. Further understanding of these relationships is important to better understand the distributed networks and their contribution to why AWS are able to speak fluently at some times and not at other times. The next step in extending these analyses is to identify the brain areas involved. By identifying the neural sources and the pattern of their interaction, neural phase coherence could be used to predict instances of stuttering. In particular, valid estimates of neural sources, obtained by controlling for the effect of volume conduction due to individual differences, will be required for a mechanistic interpretation of the idea that neural coherence taps into communication within speech motor network and potentially relates to models of speech production (Giraud and Poeppel, 2012).

Although it has been argued here that impaired sensorimotor processing primarily underlies stuttering, present findings could also be construed to support alternative explanations for disfluent speech. The involvement of the theta band could support the idea that impaired timing perception underlies stuttering Giraud and Poeppel (2012). Similarly, differences involving beta band implicate a role for cognitive functions, such as reduced attention, as contributing factors to stuttering (Ofoe et al., 2018). Altogether, maintaining fluent speech in AWS could be a multifaceted task where sensorimotor impairment is compensated by higher-order cognitive functions (Jackson et al., 2015; Bowers et al., 2018). Future lines of research could potentially tease apart relative contributions of these two factors in stuttering. Further, following the same reasoning as stated above, it can be argued that observed differences in neural phase coherence reflect compensatory strategies adopted by AWS to deal with stuttering. A resolution for this potential confound could come from investigations of neural phase coherence patterns in stuttering children who have not yet developed compensating mechanisms to offset their disfluent speech.

Our findings compliment the reports of neurological differences in AWS from resting state network differences in AWS and AWNS (Qiao et al., 2017; Ghaderi et al., 2018). The deviant speech production network(s) implicated in these studies could arguably be the same networks that elicited the altered coherence patterns prior to fluent speech onset in this study and the disfluencies reported previously (Sengupta et al., 2016). More research is clearly needed to determine if the current task-related findings can be related to the passive connectivity patterns of the resting state paradigm. Our findings add to the growing body of literature indicating speech motor planning differences in AWS. The widely referenced MEG study by Salmelin et al. (2000) is an early study that highlighted a potential anomaly in the left hemisphere during speech preparation. Very recently, Jackson et al. (2019) added to this evidence in their report that increased planning load elicited left hemisphere blood flow differences preceding fluent utterances.

The patterns of neural phase coherence in AWS and AWNS differ markedly, depending on fluent vs. disfluent speech conditions. For example, neural overactivation (Budde et al., 2014) is observed in the stuttering state that could arise from atypical theta-gamma coherence and alpha-gamma coherence in fronto-central scalp areas preceding disfluencies. In contrast, the beta band coherence with alpha and theta bands did not show any changes before disfluencies (Sengupta et al., 2017). During the fluent speech condition as reported here, however, only theta band coherences, theta-beta and theta-gamma, were found to be involved in phase coherence differences overlying centro-parietal scalp areas. It is, therefore, plausible that different brain networks are involved in maintaining fluent speech compared to networks engaged prior to and during disfluent speech. It is also plausible that more than one cortical process is engaged by the atypical networks shown herein. Persons who stutter have displayed altered inhibitory control (Markett et al., 2016) that could be mediating aspects of fluency. More work is still needed on the relationship between neural coherence and inhibition, but the possibility of multiple processes impinging on fluent speech production could be investigated within a coherence framework.

This is the first study to identify neural phase coherence differences associated with the speech planning phase preceding fluent utterances in AWS, but there are several caveats. First, the sample size was relatively low. Nevertheless, statistical analyses detected high effect sizes, and the stringent bootstrapping approach confers confidence that phase coherence-based methods could be useful in future studies of stuttering. However, a larger and more diverse sample in terms of severity and therapy history will certainly improve generalizability. Second, the stimuli, together with the experimental setting, lacks certain ecological validity. In the future, it would be desirable to extend the results reported here by incorporating sentence-level stimuli. Third, individual differences in neural organization are also reported in AWS (Wymbs et al., 2013), and will require new paradigms to capture how individual variation contributes to the stuttering trait. Lastly, a recent MEG study failed to find (Mersov et al., 2018) any significant differences in beta band power of fluent and disfluent speech of AWS. Nevertheless, the same authors did find differences in the beta band between fluent speech of AWS and AWNS. Overall, findings reported here suggest fluent speech in AWS could involve higher frequency modulations than their disfluent speech and the fluent speech of typical speakers. These findings present opportunities for understanding the transitions in neural activity that shift a speech attempt into a fluent vs. a disfluent trajectory. The marked differences among AWS that precede fluent speech provides more evidence for considering basic speech production trait difference in the pathophysiology of stuttering.

## Conclusion

In this study of neural phase coherence, pre-speech power differences between AWS and AWNS were found in the high-frequency gamma and beta bands but not the lower alpha and theta bands. It was further observed that fluent speech of AWS was characterized by decreased theta-gamma phase coherence and a corresponding increase in theta-beta coherence level. Overall, this study provides more evidence that neural phase coherence is firstly sensitive to the presence of a speech production disorder and secondly, that distinct bands can signal altered aspects of speech planning.

## Data Availability Statement

The datasets generated for this study are available on request to the corresponding author.

## Ethics Statement

The studies involving human participants were reviewed and approved by Northwestern IRB. The patients/participants provided their written informed consent to participate in this study.

## Author Contributions

RS contributed to the experimental design, tested participants, analyzed data and contributed to data interpretation, produced the figures, and co-wrote the manuscript with SN. JY contributed to the experimental design and stimulus generation and edited the manuscript. TL contributed to the experimental design and data interpretation and edited the manuscript. KP generated the experimental stimuli. VG contributed to the interpretation of the results and edited the manuscript. SN contributed to the experimental design, contributed to coding and data analyses, supported figure generation, interpreted the results, and co-wrote the manuscript with RS.

## Conflict of Interest

The authors declare that the research was conducted in the absence of any commercial or financial relationships that could be construed as a potential conflict of interest.
